# Ehlers-Danlos syndromes and their manifestations in the visual system

**DOI:** 10.3389/fmed.2022.996458

**Published:** 2022-09-27

**Authors:** Samuel Asanad, May Bayomi, Douglas Brown, Joshua Buzzard, Eric Lai, Carlthan Ling, Trisha Miglani, Taariq Mohammed, Joby Tsai, Olivia Uddin, Eric Singman

**Affiliations:** ^1^University of Maryland School of Medicine, Department of Ophthalmology & Visual Sciences, Baltimore, MD, United States; ^2^University of Pennsylvania, Perelman School of Medicine, Philadelphia, PA, United States

**Keywords:** Ehlers-Danlos syndrome, ophthalmology, visual system, ocular manifestations, eye, ocular manifestation, neurologic manifestation

## Abstract

Ehlers-Danlos syndrome (EDS) is a rare, genetically variable, heterogenous group of (currently recognized) thirteen connective tissue disorders characterized by skin hyperextensibility, tissue fragility, and generalized joint hypermobility. In addition to these commonly recognized phenotypes, recent studies have notably highlighted variable ophthalmic features in EDS. In this review, we comprehensively gather and discuss the ocular manifestations of EDS and its thirteen subtypes in the clinical setting.

## Introduction

Ehlers-Danlos syndrome (EDS) is a rare, variable group of heritable connective tissue disorders characterized by skin hyper-extensibility, tissue fragility, and generalized joint hypermobility (1). Given that connective tissue is distributed throughout the entire body, it is not surprising that EDS features multi-organ system involvement. In addition to the more commonly recognized phenotypes of EDS involving the skin and skeleton, structural and functional abnormalities affecting the eye have also been shown. This review encompasses the most recently reported ophthalmic manifestations as well as secondary visual impairments of EDS and its thirteen subtypes in clinical practice.

## Classical Ehlers-Danlos syndrome

Classic Ehlers-Danlos syndrome (cEDS) is a heterogenous group of heritable connective tissue disease. Causative gene mutations for this syndrome include either *COL5A1* or *COL5A2*, which normally code for collagen chain α1(V) and α2 (V), respectively. Abnormal coding of these genes results in structurally defective, and thus, functionally abnormal, type V collagen. Characteristic features of cEDS include hyperextensible skin, hypermobile joints, and abnormal wound healing (1, 2). In addition to these signs, various ocular findings associated with cEDS have also been reported.

### Cornea

Corneal abnormalities have been described in association with COL5A1 and COL5A2 gene mutations, coding for defective type V collagen. In particular, Giunta and Steinmann reported microcornea in patients featuring point mutations in the COL5A1 gene (3). Seveg and colleagues described abnormal corneal thinning and steepness in classical EDS patients who featured heterozygous gene mutations in either COL5A1 or COL5A1 (4). This abnormality in corneal curvature may possibly explain the frequency of myopia reported in cEDS adults (5). These corneal findings were similarly demonstrated by Villani et al. in a study of 50 classical EDS patients. Notably, Villani and colleagues also showed corneal surface irregularity and tear film dysfunction in classical EDS (6). Taken together, these corneal abnormalities may contribute to visual symptoms including myopia, astigmatism, and dry eye syndrome.

### Conjunctivae

Conjunctival abnormalities have also been documented in classical EDS. Whitaker and colleagues were the first to report severe conjunctivochalasis. Intriguingly, the patient presented with chronic foreign body sensation and ocular irritation resulting in discomfort with blinking. Surgical correction was ultimately required for the patient's symptomatic relief. The authors proposed that this conjunctival abnormality may be associated with the defective collagen V synthesis with a predisposition for elastotic degeneration from sunlight exposure and consequent hyper-elasticity (7). Such conjunctival abnormalities can present with a diverse array of visual findings including eye pain, blurry vision, excessive tearing, and redness.

### Orbit and ocular adnexa

In a study of three unrelated families with classic EDS, Segev et al. observed floppy eyelids in all subjects (4). Specifically, this eyelid abnormality was defined by excessive laxity of the upper eyelid tarsal plate that readily everts with minimal force. The proposed mechanism for floppy eyelids may be related to abnormal heterotypic type I/V collagen interactions and collagen fibrillogenesis (4). Patients with floppy eyelid syndrome may exhibit notable ocular problems including non-specific irritation and foreign body sensation, dryness, mucoid discharge, redness, eyelid edema, and photosensitivity.

Cohort studies conducted by Reitelli et al. (75 patients) and Colombi et al. (62 patients) have also reported epicanthal folds, palpebral ptosis, enophthalmos, infraorbital creases, hypotelorism, telecanthus, blue sclera, xeropthalmia, and abnormally slanted palpebral fissures in cEDS (8, 9). Notably, epicanthal folds have been more frequently observed in young patients, which then become attenuated with aging (9, 10). Patients with craniofacial abnormalities can exhibit double vision, reduced fine stereopsis and/or reduced acuity. In pediatric patients, these can lead to amblyopia and strabismus. In addition, cEDS can further be associated with dental problems that might refer pain to the orbit (11). Furthermore, cEDS rarely can be associated with CSF leak, causing headaches that may lead to neuro-ophthalmic evaluation (2).

### Vascular complications

Various vascular complications have been reported in classic EDS, which can have important visual manifestations. Among these include cervical artery dissection, which affects the blood supply to the brain and can present with various signs and symptoms including diplopia, blurred vision, transient visual obscurations, nystagmus, Horner syndrome, decreased corneal sensation and visual field defects (2). Patients with cEDS may be at increased risk for aneurysm as well (12); if occurring intracranially, they can lead to many visual problems associated with stroke.

## Classical-like Ehlers-Danlos syndrome

Classic-like Ehlers-Danlos syndrome (clEDS) is a type of EDS caused by variable mutations in the TNX (Tenascin-X) gene resulting in abnormal extracellular matrix expression in the skin, muscle, tendons, and ligaments. Inherited in an autosomal recessive manner, clEDS is characterized by joint hypermobility, skin hyper-extensibility, and vascular fragility. In addition to these clinical features, ocular involvement has also been reported in clEDS (13).

### Conjunctivae

Several conjunctival abnormalities have been documented in clEDS. In a cross-sectional study conducted by Demirdas et al. on a cohort of TNX-deficient EDS, approximately 30% of individuals exhibited recurrent subconjunctival hemorrhages (14). In addition, Lindor and Bristow documented bilateral conjunctivochalasis in clEDS (13).

Such conjunctival abnormalities can present with a diverse array of visual findings including eye pain, blurry vision, excessive tearing, and redness.

### Strabismus

Ocular misalignment has also been reported in clEDS. These patients can exhibit double vision, reduced fine stereopsis and/or reduced acuity. In pediatric patients, strabismus can lead to amblyopia (14). In addition, dental involvement reported in clEDS might present as referred pain to the orbit (5).

### Vascular complications

Various vascular complications have been reported in clEDS, which can have important visual manifestations. Among these include thoracoabdominal aortic aneurysm and aneurysm involving both common ileac artery and superior mesenteric artery. This multi-system vascular fragility characteristic of clEDS could lead to arterial rupture and brain injury from hypoperfusion, from which the visual pathways would be a risk (14).

## Cardiac-valvular Ehlers-Danlos syndrome

Cardiac-valvular Ehlers-Danlos syndrome (cvEDS) is a rare subtype within the family of Ehlers-Danlos Syndromes that is gaining wider recognition as molecular studies grow more prevalent. As of May 2022, there have only been 8 molecularly-confirmed cases of cvEDS reported in the literature with several other cases described without molecular confirmation (15–21). As there are many overlapping features across EDS subtypes, mainly skin and joint hyper-flexibility, cvEDS many times, within the literature at least, is misclassified under classical or hypermobile EDS. Rarely, may it also be confused for a case of osteogenesis imperfecta, a similar disorder characterized by genetic defects in collagen synthesis (22). However, accurate diagnosis of EDS subtypes is paramount for genetic counseling and raising clinical suspicion for various comorbid conditions. Based on the 2017 international classification of EDS, cvEDS is defined as a mutation in COL1A2 resulting in the absence of pro-a2-chain of type 1 collagen leading to non-sense-mediated mRNA decay (23). While molecular identification of the mutation is necessary for diagnosis confirmation, various criteria has been identified to aid in discovery. Certain major criteria are severe progressive cardiac-valvular problems, mainly within the mitral or aortic valves, skin involvement (hyper-extensibility, atrophic scars, thin skin, easy bruising), and joint hypermobility. Various minor criteria include the presence of an inguinal hernia, pectus deformity, joint dislocations, and foot deformities (pes planus, pes planovalgus, hallux valgus) (23).

### Anterior ocular manifestations

The ocular exam in patients with confirmed cvEDS is highly variable. Phenotypic expression ranges greatly and ocular manifestations are seldomly reported in the literature. When ophthalmic findings are reported they commonly are myopia, strabismus, astigmatism, blue sclera, and eyelid abnormalities (5, 15, 19, 21, 23). In a case series of two sisters with confirmed cvEDS, Guarnieria and colleagues describe ocular manifestations of myopia and strabismus in a patient with mildly ptotic lower eyelids (which he attributes to underdevelopment of the zygomatic bones) as well as a blue-gray hue of the sclera (15). Interestingly, in the family reported by Guarnieria, the affected sister had a normal ocular exam while their father, a healthy male, had markedly dark blue sclera (15). Similarly, Pires and his colleagues report a case where they describe the patient having “large eyes” and “sunken cheeks” (19). This may refer to the eye itself or simply be explained by the known increased laxity of periorbital skin giving the appearance of larger eyes.

### Posterior ocular manifestations

Due to wide range of phenotypic expression of patients with cvEDS as defined by COL1A2 mutation, there is often misreporting of cases, especially when patients are younger and cardiac-valvular abnormalities may be less apparent. When these valvular abnormalities do appear they most notably present in the form of mitral regurgitation, mitral valve prolapse, and aortic root dilation (18). These patients may require valve replacement which carries its own potential risk of emboli to the eye and brain as described by Barthelemy et al. (and the many vision problems associated with stroke including diplopia, visual field loss, nystagmus, etc.) (24). In addition, these patients are prone to heart failure, which can cause chronic retinal ischemic perivascular lesions identifiable on ocular coherence tomography of the retina (25, 26).

## Vascular Ehlers-Danlos syndrome

Vascular EDS (vEDS), classified as type IV EDS, is a rare connective tissue disorder due to a heterozygous pathogenic variant in *COL3A1*, which encodes for type III collagen. VEDS is typically inherited in an autosomal dominant manner, although atypical biallelic inheritance patterns have also been reported (27). Individuals with vEDS are prone to developing serious vascular, gastrointestinal, and obstetrical complications, with the highest mortality risk being arterial ruptures (28). Although major primary ocular pathologies have not been frequently reported in vEDS, various associated findings in the eye have been reported as sequelae of vascular complications.

### Orbit and ocular adnexa

Clinical features of vEDS include easy bruising, thin and translucent skin, acrogeria, and joint hypermobility. Given this pronounced skin fragility, vEDS patients may be more prone to developing spontaneous periorbital ecchymoses. Characteristic facial features in vEDS include micrognathia, prominent eyes, narrow nose, thin lip vermilion. This apparent proptosis may be accompanied by various ocular signs including lagophthalmos with consequent exposure keratopathy, conjunctival hyperemia, telangiectasias along the eyelid margin, and chemosis (29, 30).

### Optic nerve

Optic nerve abnormalities have been reported in type IV EDS. Mitra et al. reported an unusual case of advanced glaucoma in a patient with typical features of type IV EDS that presented with vision loss. On exam, the patient showed ptosis, ectropion in both eyes, right sided afferent pupillary defect, and elevated intraocular pressures of 46 and 42 mmHg in the right and left eye, respectively. Fundus exam demonstrated glaucomatous optic atrophy and the patient underwent trabeculectomy in both eyes (31). The corneoscleral and uveal trabecular meshwork are mostly composed of type I, II, and IV collagen, while the juxtacanalicular meshwork and Schlemm's canal contain type III collagen (32). Given the defect in type III collagen, it is thought that patients with vEDS may be prone to increased intraocular pressures and development of open angle glaucoma (31, 33).

### Retina

Retinal abnormalities have also been described in vEDS, as vitreous hemorrhage is one of the ocular complications observed because of thin and fragile blood vessel walls in the posterior chamber. Additionally, type III collagen is found in the tissues of arterial walls, sclera, cornea, and is thought to affect the structural integrity of the eye. This may potentially explain the association with retinal tears and/or detachments in both type IV and type VI EDS (34). With various reported developments of intraoperative complications such as choroidal detachment, hemorrhage, and subsequent scarring, success of surgical repair for rhegmatogenous retinal detachments in patients with vEDS is especially low (35). In one case of a patient with type IV EDS presenting with retinal detachment accompanied by vitreous hemorrhage and ten retinal breaks, Lumi et al. reported successful repair utilizing a small-gauge vitrectomy with gas tamponade and laser photocoagulation to avoid intraoperative trauma and intensive bleeding (36).

### Vascular complications

Vascular complications are the most defining features of type IV EDS, as affected patients are born with thin and fragile tissues leading to arterial dissections or tears, hematomas, and aneurysms. Large and medium sized arteries tend to be affected more, although all areas can be affected. One common complication of vEDS is carotid-cavernous fistula (CCF) formation, caused by defective or absent type III collagen in the arterial walls (28). Pollack et al. report a landmark case of a patient with vEDS who initially presented with vision loss, proptosis, conjunctival hyperemia and chemosis, orbital pain, and ocular bruits. Cerebral angiography revealed the cause to be a CCF draining into a greatly dilated right superior ophthalmic vein. Attempted thromboembolism of the CCF resulted in multiple complications including retinal ischemia, endophthalmitis, and subsequent fatal abdominal aortic rupture (37). Additional ocular symptoms secondary to CCF that have been documented include increased lacrimation, periorbital swelling, ophthalmoplegia, relative afferent pupillary defect, episcleral vascular congestion, and superficial punctate keratopathy (37, 38).

## Hypermobile Ehlers-Danlos syndrome

Hypermobile Ehlers-Danlos Syndrome (hEDS), the most common subtype of EDS, is characterized by the presence of generalized joint hypermobility in the context of other syndromic features (39). Although hEDS is presumed to be caused by an autosomal dominant genetic mutation, no specific gene has yet been identified in the current literature (40). Diagnosis is therefore based entirely on history and clinical exam findings defined by diagnostic criteria established by the International EDS Consortium. In addition to these systemic features, ocular manifestations have also been reported in hEDS (41).

### Xeropthalmia

Dry eye is a frequently reported symptom in patients with hEDS. Ghabriya et al. demonstrated that hEDS patients had more reports of dry eye symptoms than controls and that the severity of symptoms correlated with Schirmer test findings (42). Similarly, Gauthier et al. conducted a study on 12 patients with hEDS and found dry eye symptoms present in 83% of patients with 46% of them exhibiting objective findings including punctate epithelial erosions or decreased tear break up time. Ho et al. (43) explored the relationship between postural orthostatic tachycardia syndrome (POTS) and dry eye syndrome. Notably, they found a higher prevalence of sicca complex in EDS patients with POTS dysautonomia relative to the general public (43). Although the exact mechanism is not well delineated in the current literature, one hypothesis is that an underlying autonomic dysfunction which is classically associated with EDS may manifest as impaired tear film production secondary to diminished exocrine gland function. It was proposed that tear secretion may be impaired due to abnormal extracellular matrix production in the lacrimal gland (43).

### Myopia

Gharbiya et al. illustrated evidence of pathological myopia, defined as greater than−6.0 diopters, and associated retinal abnormalities. Approximately16% of hEDS patients exhibited significant retinal atrophy and choroidal thickening compared to 0% of controls. Examination in the highly myopic eyes showed a diffuse fibrillar and beaded appearance of the vitreous (42). Similarly, Mishra and associates found higher rates of myopia in hEDS patients defined as >3.00 diopters and 88% of patients had errors between−3 and +3 diopters. Myopia in hEDS may be related to changes in the vitreous extracellular matrix and composition of the sclera (44). Chiarelli et al. showed that hEDS fibroblasts exhibited dysregulated gene expression of extra cellular matrix glycoproteins such as elastin, suggesting matrix dysfunction as a potential mechanism (45). High myopia is associated with an elevated risk of retinal detachment, choroidal neovascularization, glaucoma and macular hole, all of which can lead to severe vision loss (46). It should be noted that the literature frequently cites a concern for the presence of angioid streaks in patients with hEDS. Angioid streaks are irregular radial breaks in Bruch's membrane that predispose the retina to choroidal neovascularization and secondary vision loss. Recently, it was reported that there is likely no increased risk of identifying angioid streaks in hEDS patients (47–49).

### Ocular adnexa

Mishra et al. examined 34 patients with hEDS and found that 68% of patients had abnormal eyelids, with 41% of patients specifically exhibiting excess lid laxity. Twenty-four percent of patients had prominent horizontal folds of upper lid skin and 1 patient had congenital unilateral ptosis (44). Eyelid laxity can lead to chronic cornea irritation and chronic papillary conjunctivitis (50).

### Cornea

Although there is no documented statistically significant keratoconus in patients with hEDS compared to the general population, there are reports of increased corneal curvature amongst hEDS patients (51). It is hypothesized that these findings are due to abnormalities in the corneal extracellular matrix. This situation could create difficulty in providing satisfactory refractive correction or fitting contact lenses comfortably. In contrast to other connective tissue disorders including kEDS and brittle cornea syndrome, there is no increased risk of corneal fragility (42).

### Lens

Lens abnormalities have also been reported in hypermobile EDS. In a cross-sectional study conducted by Gharbiya et al., minor lens opacities were significantly more prevalent in young adult hypermobile EDS subjects as compared to controls (42). Further progression of lenticular opacification can be visually symptomatic from decreased visual acuity. It must be mentioned that hEDS may be at higher risk for complications of ophthalmic surgery, adding to the complexity of treating these patients (52).

### Efferent visual function

Convergency insufficiency was reported to be common in a case series of EDS patients, with 60% of patients manifesting symptoms (48). Convergence insufficiency is associated with fatigue, nausea and headache while performing near vision tasks. This can have a profound effect on school and work performance.

### Visual complications from neurologic manifestations of hEDS

Neurologic complications of hEDS are protean (53). Migraine is more common in patients with hEDS and that can be not only associated with reduced vision from aura but postdromal effects such as glare sensitivity and possibly convergence insufficiency (54). Chiari malformation has been reported as comorbid with hEDS; abnormally low cerebellar tonsillar descent can interfere with cerebrospinal fluid (CSF) hydrodynamics leading to intracranial hypertension and vision-threatening papilledema. HEDS can lead to spontaneous CSF leak, associated with visual complaints including photophobia and diplopia (55). It should also be noted that patients with hEDS appear to have less positive recovery from concussion, with longer and slower trajectories of healing. In one series of 7 patients, the poor recovery and persistent visuomotor complaints (e.g., convergence insufficiency, deficiency of smooth pursuits, saccadic deficiency and/or glare sensitivity) actually led to the heretofore undiscovered diagnosis of hEDS (56).

## Arthrochalasia type Ehler-Danlos syndrome

Arthrochalasia Ehlers-Danlos Syndrome (aEDS), also known as Ehlers-Danlos Syndrome type VII, is an autosomal dominant condition caused by heterozygous mutations in the COL1A1 and COL1A2 genes, which normally encode the pro-alpha-1 and pro-alpha-2 chains of type 1 collagen. Such mutations result in the loss of exon 6 in either gene during the pre-mRNA processing and impairs the proper formation of collagen fibrils (57). Previously, the syndrome was further subdivided into EDS Type VIIA in cases of affected alpha-chain-1 or EDS Type VIIB in cases of affected alpha-chain-2. AEDS is a very rare disorder with an unknown prevalence and a reported case number of only 49 patients worldwide (5, 57). The diagnostic criteria can be split into major and minor factors. The major criteria include congenital bilateral hip dislocation, severe generalized joint hypermobility, recurrent small or large joint dislocation and hyper-extensibility of the skin. The minor criteria include muscle hypotonia, kyphoscoliosis, radiologically mild osteopenia, tissue fragility including atrophic scars and skin prone to bruising (5, 57). The minimal clinical criteria for diagnosis is either a combination of congenital bilateral hip dislocation with skin hyper-extensibility or severe generalized joint hypermobility with at least two other minor criteria. However, diagnosis must be ultimately confirmed with molecular genetic analysis (58). Although not included in the diagnostic criteria, ocular findings have been present in several AEDS cases, primarily involving the sclera, the lens and the external eye.

### Sclera

Scleral irregularities are associated with COL1A1 and COL1A2 gene mutations, resulting in abnormal formation of type 1 collagen. In a review of 49 patients with aEDS, Brady et al. identified 3 subjects with blue sclera (5). Blue sclera results from thinning and subsequent transparency of type 1 collagen in the sclera, which increases the visibility of the underlying choroidal pigment and ultimately gives the sclera a blue tint (5). Similar scleral changes have been associated with multiple connective tissue disorders including Marfan syndrome, Osteogenesis Imperfecta, Pseudoxanthoma elasticum, and Willems De Vries Syndrome (59). Like aEDS, osteogenesis imperfecta results from genetic mutations in COL1A1 or COL1A2 and commonly presents with blue sclerae. Pirouzian et al. reported three cases of severe globe injuries due to scleral fragility in patients with osteogenesis imperfecta, with one patient suffering complete loss of vision and the other two suffering non-sight-threatening scleral perforations (60). Since type 1 collagen fibers are important structural components of both the cornea and sclera, patients with osteogenesis imperfecta are more susceptible to ocular rupture and damage in cases of trauma (59). Although there are no reported cases of this in aEDS patients, they may possess a similar risk due to the shared pathologic features of abnormal collagen and scleral thinning. Scleral thinning is also associated with increasing degrees of myopia, presence of staphyloma, and pathological myopia lesions in the sub-foveal region (61).

### Lens

Of the reported cases of aEDS, 1 patient was found to have bilateral ectopia lentis, or dislocation of the lens of the eye (62). This patient was initially evaluated at 7 months of age due to numerous musculoskeletal and systemic symptoms including hip dislocation, joint laxity and redundant skin, and she was diagnosed at the age of 14 months after biochemical analysis. The underlying pathology of this ocular manifestation of aEDS has not been studied, but ectopia lentis is the most common manifestation of Marfan syndrome and is believed to occur due to abnormal fibrillin microfibrils leading to weak zonular fibers and structural defects of the lens capsule (63). The effects of ectopia lentis varies with the degree of dislocation but with significant disruption of the zonule structure or lens, patients may develop increased lens curvature, resulting in lenticular myopia or astigmatism (64). Lens dislocation is also associated with major ocular complications including pupillary block glaucoma.

### Orbit

AEDS has also been associated with external eye findings, mainly hypertelorism and epicanthal folds (5, 58). Of 49 patients with aEDS reviewed, three had both hypertelorism and epicanthal folds (5, 62). The pathogenesis of these facial characteristics has not been well investigated, but both have been seen in other connective tissue disorders as well. Hypertelorism is a common finding in Loeys-Dietz syndrome, which most often results from variants of the SMAD and TGFB genes and can impact genetic products such as collagen (65). Epicanthal folds are also seen in other subtypes of Ehlers-Danlos syndrome including classical (EDS types I and II) and dermatosparaxis (EDS type VIIC). The repair of hypertelorism improves patient appearance and it would seem likely self-esteem (2, 5, 65). Recognizing that patients with EDS report a negative psychosocial impact from their medical condition, it would seem reasonable that facial deformities might only worsen this situation.

## Dermatosparaxis Ehlers-Danlos syndrome

Dermatosparaxis Ehlers-Danlos syndrome (dEDS) is an extremely rare subtype of EDS with only 10 reported cases (66). Characteristic features include severe skin fragility and laxity, sagging redundant skin, facial gestalt, excessive bruising, and complications secondary to visceral and vascular fragility such as large hernias (66, 67). DEDS is caused by an autosomal recessive mutation in ADAMTS2 (A Disintegrin-like And Metalloprotease domain with Thrombospondin type 1 motifs 2), an enzyme that cleaves the aminoterminal propeptide domain of types I, II, and II procollagen (66). In addition to these signs, various ocular findings associated with dEDS have also been reported.

### Cornea

Corneal abnormalities have been described in association with the ADAMTS2 gene mutation, coding for a defective metallopeptidase. Foster et al. reported a decrease in ADAMTS2 expression in cultured keratoconus cells (68). ADAMTS2 mutations have also been implicated in myopia in genome-wide association studies by Flitcroft et al. (69). These corneal abnormalities can together contribute to various symptoms including blurred or distorted vision, glare, photosensitivity, and difficulty with driving at night.

### Lens

Hereditary ectopia lentis or lens dislocation has been shown to have to have genetic link to mutations in the ADAMTS family (70). Consequently, dEDS patients may not only exhibit refractive shifts, but also the potential complications of crystalline lens malposition including pupillary block glaucoma, retinal damage, and even blindness.

### Orbit and ocular adnexa

Reports by Malfait et al. and Colige et al. have reported ocular features such as blue sclera and puffy eyelids (67, 71). DEDS has also been associated with various dental abnormalities such as micrognathia, a frontal open bite, gingival hyperplasia, obliteration of tooth pulp, severe enamel attrition, dysplastic roots, which may increase risk for referred pain to the orbit (67).

### Vascular complications

Dermatosparaxis has also been implicated to have a higher risk of intracranial hemorrhages (18% of non-vascular subtypes cases with vascular phenotypes) (72). Intracranial hemorrhages, if occurring in the visual pathway, can lead to visual field defects and ocular movement deficits.

## Kyphoscoliotic Ehlers Danlos syndrome

Kyphoscoliotic Ehlers Danlos Syndrome (kEDS) is a sub-type of EDS caused by autosomal recessive mutations in the PLOD1 gene which encodes the enzyme lysyl hydroxylase 1, or the FKBP14 gene which encodes the FK506 22 kDa binding protein. These mutations ultimately cause impairments in procollagen folding in the endoplasmic reticulum and in collagen cross-linking (73). This subtype is rare, with an estimated prevalence of 1 in 100,000 (74). Clinical manifestations of kEDS include early onset kyphoscoliosis, joint hypermobility, muscle hypotonia, and fragility of the skin and vasculature throughout the body (23). In addition to these findings, specific ocular abnormalities are often reported in patients with kEDS. The unifying theme of these ocular findings is in fragility of the affected structures, leading to increased risk of rupture and damage.

### Corneal abnormalities and refractive errors

Microcornea, i.e., abnormally small corneal diameter, has been reported in patients with kEDS; it is one of the minor criteria for the diagnosis of PLOD1 gene-specific kEDS (23). In a case series of six patients with confirmed PLOD1-related kEDS, all of the patients had microcornea, with cornea diameters ranging from 9 to 10.5 mm (75). Notably, normal adult cornea diameter ranges from 11.04–12.50 mm in men and 10.70–12.58 in women. In a separate case series of eleven patients with kEDS, all patients demonstrated corneal thinning, a substantial risk for corneal swelling and rupture (75). Indeed, seven of the eleven patients evaluated had experienced corneal rupture spontaneously or after minimal trauma, and one had corneal hydrops.

Abnormalities in corneal size and contour may also cause refractive errors, another problem on the list of minor criteria for the diagnosis of kEDS (23). In one case series, ten out of eleven patients with kEDS had mild to severe myopia (76). Ni and colleagues report a case of an individual diagnosed with kEDS at the age of 17 who developed permanent visual acuity reduction from refractive amblyopia by age 3 years (77). This highlights the importance of early assessment for refractive error in children, and of considering diagnoses like kEDS when multisystem manifestations of connective tissue dysfunction are present.

### Ocular fragility and globe rupture

Blue sclerae are commonly reported in patients with kEDS. The differential for blue sclerae is broad, including connective tissue disorders like osteogenesis imperfecta, Marfan syndrome, Van der Heave syndrome, and EDS. However, blue sclerae in particular are a minor criteria for diagnosis of kEDS (23, 74, 75, 77). This finding occurs because the collagenous sclera is thin, allowing visualization of underlying uvea. Aside from the cosmetic implications of blue sclera, scleral thinning elevates the risk for globe rupture in patient with kEDS, and rupture can occur spontaneously (78). Furthermore, repair of globe rupture in patients with scleral thinning is expected to be less successful in saving vision. Finally, the diagnosis of globe rupture in the absence of trauma or after trivial trauma should prompt a consideration of a diagnosis of underlying connective tissue disease.

### Vascular fragility

Blood vessel fragility is a hallmark of multiple EDS subtypes, including kEDS (23, 74). This fragility may extend to retinal vessels, increasing the risk of sight-threatening retinal or vitreous hemorrhage and retinal detachment (34). Patients with kEDS are also at elevated risk of arterial dissection or perforation as well as perinatal stroke. Stroke affecting the visual pathways can be manifest as a wide range of problems including visual acuity and field loss, strabismus and nystagmus.

## Brittle cornea syndrome

Brittle Cornea Syndrome (BCS) is a grouping of two known genetic mutations, which result in significant thinning of corneal tissue (220–450 microns) due to dysregulation of extracellular matrix regulation. Type 1 and Type 2 BCS result from mutations of the zinc finger protein-469 (ZNF469 - chromosome 16q24) and PR domain-containing protein (PRDM5/PFM2 – chromosome 4q27) genes, respectively (79, 80). Both affected genes are associated with maintenance and generation of normal corneal thickness, while PRDM5 is also linked to retinal development. Both genes assist with extracellular matrix regulation at the transcription level. Various studies have shown their misfunction is associated with deleterious effects on collagen regulation, cell migration/adhesion, and development and maintenance of extracellular matrix components (80–82). Although predominantly involving the eyes, systemic features of BCS including hyper-extensible skin, hypermobile joints, hyper-compliant tympanic membranes, hearing loss, joint contractures and arachnodactyly have been identified (23). Various ocular structures are impacted by BCS, resulting in a constellation of symptoms.

### Cornea

BCS primarily affects the cornea, with corneal thinning manifesting as progressive, early onset keratoglobus or keratoconus. This thinning results in fragility first described in 1968 by Dr. Richard Stein, after attempted suture repair of a corneal perforation resulted in the cornea breaking into “crumbs” (83). Due to this thinning, patients are at high risk for corneal rupture and perforation with minor trauma, often at a young age. Given the fragility of the corneal tissue, perforations in these patients are quite difficult to treat, and infection risk is elevated post-operatively, potentially leading to blinding infections (84). In addition, patients with BCS experience high myopia, megalocornea, and corneal scarring. The myopia is associated with normal to slightly elevated axial lengths and is thought to be due to corneal thinning and steepening. Corneal scarring often presents as a chronic result of keratoconus or related to previous repair attempts (23, 80). As BCS usually causes corneal breakdown and subsequent damage and scarring early in life, children are at serious risk to develop amblyopia (85). The challenging treatment of this condition is illustrated by Shalicka et al. in their report of long term follow up for patient with ZNF469 mutation; despite best efforts and timely management of the patient's corneal rupture and complications, the patient was left with light perception vision in just one eye and became blind in the other (86). Notably, carriers of BCS genetic mutations are generally asymptomatic, but may also display myopia and some degree of corneal thinning (81). Altogether, patients with BCS have thin, fragile corneas predisposed to traumatic damage after minor trauma, often presenting a surgical challenge, with associated infection risk, refractive error and structural corneal changes.

### Conjunctivae and sclera

The association of blue-tinged sclera exists in various conditions including the Ehlers-Danlos Syndromes (BCS included), Marfan syndrome and osteogenesis imperfecta (87). Although blue sclera is not unique to, nor universally present in patients with BCS, it can provide useful clinical information. Presence of blue sclera correlates with at least a 33% reduction in central corneal thickness (79). Though non-specific to BCS, presence of blue sclera can suggest corneal thinning and should prompt additional investigation in any case.

### Retinal detachment and glaucoma

Rare features of BCS may include retinal detachments and possible secondary glaucoma. These cases are sparsely reported in the literature in a few cases and are often reported in association with high myopia (88, 89). Notably, experts emphasize that retinal detachment and glaucoma is rare in BCS, and usually are not findings seen at initial presentation, though their incidence is possibly masked by need for management of globe perforations at early ages, as well as by enucleation or other complicating factors (80, 81).

## Spondylodysplastic Ehlers Danlos syndrome

Spondylodysplastic Ehlers Danlos Syndrome (spEDS) is a type of EDS that is characterized by short stature, muscle hypotonia, and bowing of the limbs (23, 90). There are 3 gene-specific minor criteria associated with connective tissue development in spEDS including *B4GALT7* (encodes galactosyltransferase I), *B3GALT6* (encodes galactosyltransferase II), and *SLC39A13* (encodes homodimeric transmembrane zrt/irt-like protein 13 or ZIP13 (23, 90). Notable ophthalmic findings associated with these genes have been reported.

### Anterior eye anomalies: Cornea, sclera, iris, and lens

Various anterior eye abnormalities have been documented in spEDS. Arunrut et al. observed bilateral corneal clouding with unilateral corneal scarring in a patient with a missense mutation in *B4GALT7* (91). Corneal clouding can make the ophthalmic exam especially challenging given a difficult view of the lens, retina, and optic nerve. Studies conducted by Van Damme et al., Cartault et al., Sellars et al. have shown microcornea, megalocornea, and sclerocornea in spEDS, respectively (92–94). These corneal abnormalities in spEDS may be related to decreased dermal and corneal stromal collagen as demonstrated by Fukada et al. in *Slc39a13* knockout mice (95). These corneal changes can not only cause visual symptoms including myopia and hyperopia, but also increase the risk of glaucoma.

Arunrut et al. observed bilateral inferonasal iris colobomas in a 5-year-old female with a novel missense mutation in *B4GALT7* (91). Experiments conducted by Bullock et al. demonstrated iris colobomas and cataracts in mice homozygous for a gene trap allele targeting heparan sulfate-2 sulfotransferase (*Hs2st1*) (96). Several groups have also illustrated lens abnormalities in spEDS including posterior subcapsular cataracts (91, 97, 98). Munns et al. reported a case of bilateral posterior subcapsular cataracts in a 12-year-old with spEDS who was treated with bilateral lensectomy. Several investigators have also documented blue sclera in spEDS (90, 99–101).

### Orbit and ocular adnexae

Characteristic craniofacial features include frontal bossing, mid-face hypoplasia, triangular facies, prominent eyes or proptosis, down-slanting palpebral fissures, strabismus (esotropia, exotropia), and congenital ptosis (91). Patients with craniofacial abnormalities can exhibit double vision, reduced fine stereopsis and/or reduced acuity. Furthermore, these can lead to strabismus (esotropia, exotropia), which in pediatric patients, can result in amblyopia.

### Posterior eye anomalies: Optic nerve and retina

Posterior eye abnormalities have also been documented in spEDS. Peripapillary atrophy as well as optic nerve atrophy secondary to glaucoma have been shown (90). Additional studies have revealed bilateral small optic nerves and bilateral optic nerve coloboma (91). Experiments conducted by Cai et al. in mice have demonstrated defective optic disc and stalk development associated with mutations in the heparan sulfate (HS) N-sulfotransferase genes (*Ndst1* and *Ndst2*) and HS O-sulfotransferase genes (*Hs2st, Hs6st1*, and *Hs6st2*) cause (102). Similarly, Fuerst et al. showed optic stalk coloboma secondary to overexpression of agrin, a major HS proteoglycan in the internal limiting membrane in mice (103).

In addition to the optic nerve, retinal pathology has also been shown in spEDS. Malfait et al. and Munns et al. observed spontaneous retinal detachments in patients with B3GALT6 mutations (90, 97). Funduscopic studies conducted by Alanay et al. revealed pigmentary retinopathy and retinal vascular attenuation in patients with spondylo-ocular syndrome associated with mutations in XYLT2 (104).

## Musculocontractural Ehlers-Danlos syndrome

Musculocontractural Ehlers-Danlos Syndrome (mcEDS) is rare subtype of Ehlers-Danlos that has been reported in fewer than 100 patients worldwide (105, 106). McEDS is autosomal recessive and is caused by a deficiency in the glycosaminoglycan dermatan sulfate (107). This deficiency results from a defect in either the carbohydrate sulfotransferase 14/dermatan 4-O-sulfotransferase 1 gene (CHST14/D4ST1) or dermatan sulfate epimerase (DSE) gene (105). The relative absence of dermatan sulfate leads to an excess of chondroitin sulfate which ultimately disrupts the normal structure of collagen throughout the body. Ocular manifestations vary between mcEDS-CHST14 and mcEDS-DSE and their associated mutations. Common ocular manifestations including strabismus, glaucoma, and refractive error are listed as minor criteria for mcEDS (23).

### Refractive error

MCEDS patients may present with visually significant refractive errors. Among these, myopia is most commonly reported followed by astigmatism, and hyperopia (23, 108–110). In turn, mcEDS patients may complain of blurry vision requiring appropriate spectacle correction. Correction of high refractive errors are especially important early in life to avoid amblyopia.

### Cornea, sclera, iris

Anterior segment anomalies have been reported in mcEDS. For example, microcornea, i.e., abnormally small cornea (<10 mm diameter) associated with a crowded anterior segment can be demonstrated (110). Microcornea is associated with a number of ocular comorbidities including lens maldevelopment with cataract formation and angle closure glaucoma. Sclera findings present in mcEDS include pannus, neovascularization, cornea plana, sclerocornea, scleromalacia, and blue sclera (106, 108). These conditions can lead to reduced vision, amblyopia, increased risk of ocular perforation after trauma and increased likelihood of requiring corneal transplant to rehabilitate vision.

Jenecke et al. have reported iris atrophy and posterior as well as anterior synechiae in mcEDS, raising the risk of glaucoma. Developmental eye abnormalities have also been observed in mcEDS including nanophthalmos, in which the entire eye is smaller than normal, or microphthalmos where the eye is small and disorganized (108). Both structural abnormalities elevated the risk of angle closure glaucoma.

### Orbital and ocular adnexae

Primary defects of mcEDS can affect many ocular and orbital structures. Patients with mcEDS have a characteristic appearance resulting from craniofacial defects, leading to malar hypoplasia, downward slanting eyes, and hypertelorism (105, 106, 110). Other oculofacial abnormalities can include bushy eyebrows with or without synophrys, proptosis, and ptosis (106, 108). These dysmorphisms can negatively impact patient self-esteem and psychological health, already known to be suboptimal in patients with EDS (111). In the setting of craniofacial abnormalities, mcEDS may also exhibit significant extraocular muscle misalignment or strabismus (23, 110). In turn, patients can complain of diplopia and if not corrected early in life, amblyopia may ensue.

### Retina

Various retinal abnormalities have also been reported in mcEDS. These include retinal hemorrhages, peau d'orange fundus, as well peripheral retinal atrophy (106). In some reports, loss of vision from mcEDS was caused by retinal degeneration and detachment, and lacquer cracks with macular bleeding (106, 109).

### Optic neuropathy

Glaucoma with elevated intraocular pressure is frequently reported in mcEDS. As a chronic progressive optic neuropathy, glaucoma should be diagnosed and treated early to prevent irreversible loss of vision. MCEDS patients can present with either open- or closed-angle glaucoma, the latter of which may be associated with anterior segment abnormalities reported above (23).

### Vascular complications

Patients with mcEDS commonly suffer neuromuscular and neurodevelopmental delay (106). Due to easy bleeding, patients are at higher risk for the development of intracerebral hemorrhage, which can be associated with visual field- and acuity loss, strabismus and nystagmus. Epidural bleeding and secondary obstructive hydrocephalus has also been reported after minor trauma, which can lead to papilledema and secondary permanent optic nerve damage (109).

## Myopathic Ehlers-Danlos syndrome

Myopathic Ehlers-Danlos Syndrome (mEDS) is a rare subtype of EDS caused by COL12A1 gene mutation resulting in the abnormal production of collagen type XII-alpha chain, affecting myomatrix structure and function. In contrast to other EDS subtypes, mEDS can be inherited through either an autosomal dominant or autosomal recessive pattern (23). Clinical features of mEDS include congenital muscle hypotonia and atrophy that often improves with age, developmental motor delay, hypermobility, proximal joint contractures, soft skin, and atrophic scarring (65, 107).

Ocular manifestations in myopathic EDS have been described. Symptoms associated with motor developmental delay and myopathy include muscle weakness, stiffness, cramps, and spasms (107). These might be manifest as ptosis, painful blepharospasm, and intermittent diplopia, possibly leading to amblyopia. In addition, myomatrix pathology seen in mEDS can potentially compromise eyelid structural integrity resulting in excessive eyelid laxity. In turn, patients may complain of chronic foreign body sensation, dry eyes, eye redness, and epiphora from impaired lacrimal pump function secondary to abnormal eyelid closure. Furthermore, atrophic scarring characteristic of mEDS has important implications regarding ophthalmic surgical candidacy given impaired postoperative wound healing. Since muscle weakness and atrophy decreases with age, it may sometimes be prudent for practitioners to delay orbital and/or periorbital surgical repair to optimize postoperative outcomes.

## Periodontal Ehlers-Danlos syndrome

Periodontal Ehlers-Danlos syndrome (pEDS) is a rare subtype of EDS with autosomal-dominant inheritance caused by mutations in two linked genes, C1R and C1S, which encode complement 1 subunits C1r and C1s. PEDS is characterized by early-onset periodontitis and loss of teeth, joint hypermobility, and variable skin findings (112). Notably, ophthalmic abnormalities have also been reported in pEDS.

### Orbit and ocular adnexae

Prominent eyes and Marfanoid facial features such as widely spaced eyes (hypertelorism) enophthalmos, down-slanting palpebral fissures have been documented in pEDS (112). These dysmorphisms might contribute to reduced self-esteem and add to the psychologic pain widely reported in EDS patients (113). These individuals may exhibit notable ocular problems including non-specific irritation and foreign body sensation, dryness, mucoid discharge, redness, *eyelid* edema, and photosensitivity. PEDS has also been associated with an overall increased rate of infection. In the setting of sinus infections, pEDS patients may at an increased risk for orbital cellulitis which can present with eye pain, restricted ocular motility, periorbital swelling, and in severe cases, decreased color vision followed by complete loss of vision secondary to optic nerve compression.

Almost all individuals with pEDS (96%) reported easy bruising from early childhood (114). Skin fragility involving the periorbita can result in significant dermatochalasis and related visual symptoms including crowding of visual field, eyelid heaviness and associated headache by end of day (114–116). In addition, atrophic scarring delayed wound healing has important implications regarding ophthalmic surgical candidacy given impaired postoperative wound healing.

### Vascular abnormalities

Various vascular complications have been documented in pEDS including aortic dissection, rupture of cerebral aneurysm, and myocardial (117, 118). In these circumstances, it may be worthwhile counseling pEDS patients to avoid various activities including high-impact sports and weightlifting. In addition, brain MRI in adults with pEDS have shown white matter abnormalities consistent with underlying small-vessel disease associated with aging (117, 118). Such patients with intracranial vasculopathy may experience transient monocular loss of vision, which should prompt an immediate stroke work up. Furthermore, patients with cerebrovascular disease affecting the visual pathways may develop visual field deficits, nystagmus, and strabismus.

Table 1 cross-tabulates the diverse array of ophthalmic manifestations across the thirteen EDS subtypes.

**Table 1:** Ophthalmic manifestations of the 13 Ehlers Danlos Syndrome subtypes.

	**Classical**	**Classical-like**	**Cardiac-valvular**	**Vascular**	**Hypermobile**	**Arthrochalasia**	**Dermatosparaxis **	**Kyphoscloiotic**	**Brittle cornea syndrome **	**Spondylodysplastic**	**Musculocontractural**	**Myopathic**	**Periodontal**
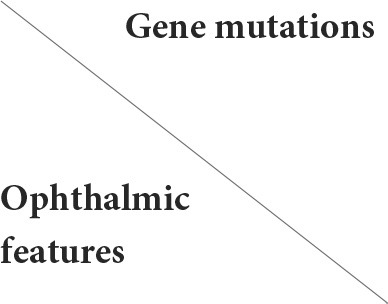	**COL5A1**, **COL5A2**	**TNX**	**COL1A2**	**COL3A1**	**Unknown**	**COL1A1, COL1A2**	**ADAMTS2**	**PLOD1**, **FKBP14**	**ZNF469**, **PRDM5/** **PFM2**	**B4GALT7**, **B3GALT6**, **SLC39A13**	**CHST14/** **D4ST1**, **DSE**	**COL12A1**	**C1R**, **C1S**
**Cornea**								✓		✓	✓		
Microcornea	✓									✓			
Megalocornea										✓	✓		
Sclerocornea													
Tear film dysfunction	✓				✓								
Astigmatism			✓					✓	✓				
Corneal thinning	✓				✓		✓						
Exposure keratopathy				✓									
**Conjunctivae**													
Conjunctivochalasis	✓	✓											
Subconjunctival hemorrhage		✓													
Conjunctivitis					✓								
Chemosis				✓									
**Sclera**								✓	✓		✓		
Blue sclera	✓		✓			✓	✓				✓		
Scleromalacia													
**Iris**										✓			
Iris coloboma											✓		
Iris atrophy											✓		
Synechiae													
**Lens**										✓			
Lenticular opacification					✓								
Lens dislocation						✓	✓						
**Orbit**													
Referred orbital pain												✓	
Proptosis/prominent eyes				✓								✓	
Enophthalmos	✓												
**Ocular Adnexae**												✓	
Floppy eyelids/excess lid laxity	✓		✓		✓							✓	
Ptosis	✓		✓	✓	✓							✓	
Blepharospasm				✓									
Ectropion				✓						✓	✓		✓
**Facial Dysmorphism**	✓					✓					✓		
**Strabismus**	✓	✓	✓		✓								
**Retina/Vitreous/Choroid**								✓	✓	✓	✓		
Retinal tears/detachment				✓	✓			✓			✓		
Vitreous hemorrhage				✓									
Macular hole													
Angioid streaks					?								
Choroidal neovascularization					✓								
Choroidal detachement				✓						✓			
Pigmentary retinopathy													
**Optic Nerve**									✓	✓	✓		
Glaucoma				✓						✓			
Optic nerve coloboma											✓		
Papilledema					✓								
**Eye Globe**											✓		
Microphthalmos											✓		
Nanophthalmos								✓	✓				
Globe rupture						✓							
**Neurovascular Complications**													
Horner syndrome	✓							✓			✓		✓
Cerebrovascular accident	✓	✓	✓	✓			✓						
Carotid-cavernous fistula				✓									

## Discussion

The spectrum of connective tissue pathology across the EDS subtypes has notable multi-system clinical features, which extend to the eye and visual pathways. The current article reviews the wide range of ocular abnormalities among the thirteen EDS subtypes and discusses their clinical manifestations together with associated symptomatology as may be encountered by medical practitioners. Early diagnosis of EDS is important to reduce the associated comorbidities of visual impairment. EDS is diagnosed through a combination of clinical findings and established clinical criteria in conjunction with confirmatory molecular genetic testing. Nevertheless, diagnosis is often delayed as the clinical findings for these disorders are often subtle and the clinical criteria continues to evolve. This is complicated by the fact that hEDS, likely the most common subtype, has no identifiable genetic marker to date. Considering these diagnostic challenges, the true prevalence of many of these disorders is unclear. The precise pathophysiology of some of the ophthalmic features is unknown and future investigations on ultrastructural intracellular characteristics may provide valuable insight into molecular pathogenesis. In addition, longitudinal studies together with epidemiologic studies surveying the ocular anomalies in EDS and their diverse clinical course among the subtypes are necessary for delineating clinical practice guidelines. An improved understanding of EDS may ultimately provide new perspectives for diagnosis and treatment of the various ocular manifestations in addition to permitting timely interventions and reasonable accommodations for improved quality of life.

## Author contributions

SA, MB, DB, JB, EL, CL, TMi, TMo, JT OU, and ES contributed to the generation of the concept and writing and editing of this review. All authors contributed to the article and approved the submitted version.

## Conflict of interest

The authors declare that the research was conducted in the absence of any commercial or financial relationships that could be construed as a potential conflict of interest.

## Publisher's note

All claims expressed in this article are solely those of the authors and do not necessarily represent those of their affiliated organizations, or those of the publisher, the editors and the reviewers. Any product that may be evaluated in this article, or claim that may be made by its manufacturer, is not guaranteed or endorsed by the publisher.
